# Ossification of the Whole Muscular System

**Published:** 1870-08

**Authors:** 


Ossification of the whole Muscular System.—A curious case of this sort is reported by Dr. Byers, of Texas. A healthy child of eight years had small tumors appear on the surface of his body; they gave no pain, and in a year and a-half disappeared, but were followed by anchylosis of the joints and a slow deposit of ossific matter in the muscles. Nearly all the voluntary muscles became hard and useless. He did not increase in stature after his tenth year, and never manifested signs of virility. He died at seventeen, never having suffered pain. The language of Dr. B. is such, that we are left in doubt as to whether the deposit was really a calcification, a deposit of earthy salts, or whether it had the characteristics of true bone tissue.—*Ibid.*

